# An investigation into the psychological status and influencing factors among residents undergoing standardized training

**DOI:** 10.3389/fmed.2025.1629789

**Published:** 2025-08-15

**Authors:** Zhenhua Liu, Haoyuan Zhang, Yufei Zhang, Lifang Zhou, Yixuan Wu, Zihan Lang, Leyuan Zhang, Haoyu Zhang, Qianqian Yu

**Affiliations:** ^1^School of Public Health, Shandong Second Medical University, Weifang, Shandong, China; ^2^Department of Social Medicine and Health Management, School of Public Health, Cheeloo College of Medicine, Shandong University, Jinan, China; ^3^NHC Key Lab of Health Economics and Policy Research (Shandong University), Jinan, China; ^4^Center for Health Management and Policy Research, Shandong University (Shandong Provincial Key New Think Tank), Jinan, China; ^5^Department of Hospital Infection Management, Shandong Provincial Third Hospital, Jinan, China; ^6^School of Management, Shandong Second Medical University, Weifang, Shandong, China

**Keywords:** mental health, standardized residency training, Symptom Checklist-90, occupational stressors, influencing factors

## Abstract

**Background:**

Physician mental health has become an increasingly urgent concern worldwide, yet little attention has been paid to the psychological wellbeing of resident physicians in China. This study aims to assess the mental health status of Chinese resident physicians and identify key demographic and professional factors associated with psychiatric symptoms.

**Methods:**

A cross-sectional survey was conducted among 276 resident physicians at Qilu Hospital, Shandong University. Participants completed a structured questionnaire that included demographic information and the Symptom Checklist-90 (SCL-90) to evaluate psychological distress. Residents were classified as screening positive for psychiatric symptoms if they met any of the following criteria: a total score ≥160, more than 43 items rated ≥1 (mild), or at least one item rated ≥3 (moderate). Statistical analyses included independent-sample t-tests, chi-squared tests, and binary logistic regression to identify influencing factors.

**Results:**

Among 276 respondents, 20.1% screened positive for psychiatric symptoms. Compared to the Chinese general population, residents showed significantly elevated scores in obsessive symptoms (*p* < 0.001), depression (*p* = 0.029), anxiety (*p* < 0.001), hostility (*p* < 0.001), phobic anxiety (*p* < 0.001), and paranoia (*p* = 0.007). Logistic regression analysis revealed that longer training years (OR = 2.24, *p* = 0.011) and having a partner (OR = 48.44, *p* < 0.001) were associated with higher odds of psychiatric symptoms. Conversely, urban residence (OR = 0.15, *p* < 0.001), being an only child (OR = 0.35, *p* = 0.020), and holding a physician’s license (OR = 0.15, *p* < 0.001) were protective factors. No significant associations were observed for gender, education level, training identity, or household monthly income.

**Conclusion:**

Chinese resident physicians experience a high burden of psychological distress, with multiple risk and protective factors identified. These findings highlight the need for tailored mental health interventions, including improving working conditions, strengthening professional support systems, and addressing interpersonal and career-related pressures during residency training.

## Background

As we all know, mental health issues among doctors are both common and serious worldwide. In March 2021, the Institute of Psychology of the Chinese Academy of Sciences conducted a survey of 2,466 medical workers in China (1). The results showed that 27.7% of medical workers may be prone to depression, with doctors identified as the highest-risk group (high risk of depression ranging from 5.1 to 12.8%), followed by nurses (high risk of 9%). According to the Medscape Physician Suicide Report 2023, which analyzed data from over 9,100 physicians across 29 specialties, 10% reported having thoughts of suicide, and 1% had attempted suicide (2). Unfortunately, mental health issues among doctors have not received widespread attention in China, especially not among resident physicians.

Mental disorders can undermine residents’ professional development, place patients at risk, and contribute to a variety of adverse personal consequences, including suicidal ideation and substance abuse (3–8). Given their close contact with patients, residents have significant social responsibility and face considerable economic pressure, which can negatively affect their physical and mental health (3, 9–12). However, few residents seek help due to a lack of early identification and access to treatment (13, 14). A prospective study showed that inadequate nightly rest was associated with a higher incidence of surgical complications among residents (15). Quantitative analyses of quality of life, combined with psychometric assessments, can increase understanding of residents’ overall health status, contribute to research on health-related quality of life, and provide basic data for health economics evaluations. We aim to explore the health status of residents and investigate the influencing factors to provide suggestions for improving their quality of life, so that they can actively work in a good physical and mental state, reduce the probability of medical errors, protect the stability of the medical system, and build a harmonious doctor–patient relationship.

Therefore, we hypothesized that the prevalence of psychiatric symptoms among Chinese resident physicians is significantly influenced by personal and professional characteristics, with longer training years and being in a relationship increasing psychological burden, while urban residency, only-child status, and physician licensure may confer protective effects.

## Methods

Residents who had worked at least 1 year in Qilu Hospital of Shandong University were considered eligible for our study. Those who had finished residency or had not worked for at least half a year were excluded from this study. The questionnaires were sent by the Medical Training Office of Qilu Hospital. A total of 278 questionnaires were distributed, and 276 valid questionnaires were retrieved, with a response rate of 93.9%. Informed consent was obtained from all participating residents.

A self-designed questionnaire was used to investigate residents of standardized training. The questionnaire included two parts: the general condition questionnaire and the psychological test scale. The general condition questionnaires included items on gender, training years (continuous), residence, being married, only child (a resident physician who grew up as the sole child in their household), physician’s license, education background, training identity, and household monthly income (continuous). Psychometric symptoms were assessed using the Symptom Checklist-90 (SCL-90), which has 90 items, including a wide range of psychiatric symptomatology, from feelings, emotions, thinking, consciousness, behaviors, living habits, interpersonal relationships, diet, and sleep. The scale has nine subscales, including somatization, obsessive symptoms, interpersonal sensitivity, depression, anxiety, hostility, terror, paranoia, and psychosis. Each item was rated on a 5-point Likert scale ranging from 1 to 5, corresponding to symptom severity: asymptomatic, mild, moderate, moderately severe, and severe. The total score ranged from 90 to 450, with higher scores indicating greater symptom severity. In this study, participants were classified as screening positive if they met any of the following criteria: a total score ≥160; more than 43 items rated as mild or above; or at least one item rated as moderate or above. The results were compared with normative data from the Chinese general population (16).

To improve the effectiveness of data collection, this study was conducted using an online questionnaire hosted on Wenjuanxing, a web-based survey platform. Before beginning the questionnaire, participants were provided with instructions explaining the general scoring methods and response requirements. All residents completed the self-assessment independently. To ensure data accuracy, the questionnaire could not be submitted if any items were left unanswered.

### Statistical analysis

All questionnaire responses were entered into an EpiData 3.1 database using a double-entry method to ensure data accuracy. Continuous variables were summarized as means and standard deviations (SDs), while categorical variables were described using frequencies and percentages. Group differences in demographic characteristics by psychiatric symptom status were assessed using independent-sample t-tests or chi-squared tests, as appropriate. Binary logistic regression was performed to identify factors associated with mental health outcomes (17). Given the multiple comparisons involved in testing several predictors simultaneously, this analysis should be interpreted as exploratory rather than confirmatory. Benjamini–Hochberg correction was applied to control the false discovery rate (FDR) at 5%. All statistical analyses were conducted using Stata, with a two-sided *p*-value of <0.05 considered statistically significant (17).

## Results

### General condition of the involved residents

**Figure 1:**
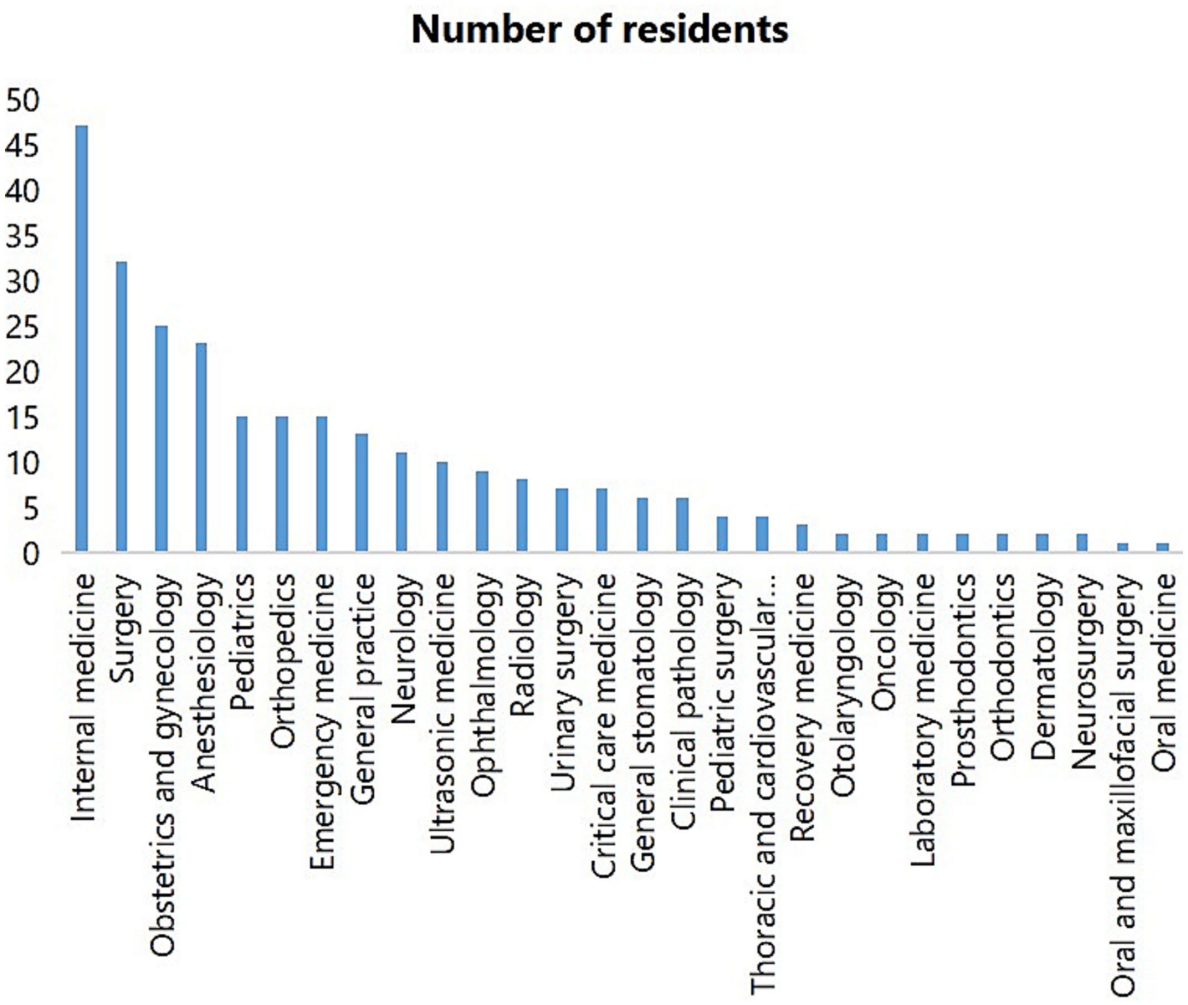
Number of residents in training specialties.

A total of 278 resident physicians from Qilu Hospital of Shandong University were included in this study. Among them, 56 (20.1%) were screened positive on the SCL-90 scale (Table 1). The prevalence of psychiatric symptoms varied significantly across several demographic and professional variables. Residents without a physician’s license exhibited a significantly higher rate of SCL-90 positivity compared to those who were licensed (27.3% vs. 14.2%, *p* = 0.007). Similarly, professional master’s trainees were more likely to screen positive than their non-professional master’s counterparts (28.0% vs. 14.6%, *p* = 0.006). Having a partner was associated with a markedly higher positivity rate compared to those without a partner (32.7% vs. 1.8%, *p* < 0.001). In terms of family background, non-only children had a significantly higher prevalence of psychiatric symptoms compared to only children (26.1% vs. 12.2%, *p* = 0.005). Residents living in rural areas were also more likely to screen positive than those in urban settings (40.2% vs. 10.3%, *p* < 0.001). Furthermore, the mean number of training years was significantly greater among residents with psychiatric symptoms (1.57 ± 0.81) compared to those without (1.12 ± 0.44; *p* = 0.006). In contrast, no statistically significant differences were observed for education level (graduate vs. bachelor’s degree, *p* = 0.363) or gender (female vs. male, *p* = 0.061), although the latter approached the threshold for significance. Residents covered more than 30 specialties, including internal medicine, surgery, obstetrics and gynecology, anesthesiology, and pediatrics. The number of residents of each specialty is shown in Figure 1.

**Table 1:** General condition of involved residents.

Variable	SCL-90 Negative (*n* = 220)	SCL-90 Positive (*n* = 56)	Total (*n* = 276)	*p*-value
Physician’s license				0.007
No	93 (72.7%)	35 (27.3%)	128	
Yes	127 (85.8%)	21 (14.2%)	148	
Education level				0.363
Bachelor’s degree	144 (81.4%)	33 (18.6%)	177	
Graduate degree	76 (76.8%)	23 (23.2%)	99	
Training identity				0.006
Non-Professional Master	135 (85.4%)	23 (14.6%)	158	
Professional Master	85 (72.0%)	33 (28.0%)	118	
Being married				<0.001
No	109 (98.2%)	2 (1.8%)	111	
Yes	111 (67.3%)	54 (32.7%)	165	
Only child				0.005
No	119 (73.9%)	42 (26.1%)	161	
Yes	101 (87.8%)	14 (12.2%)	115	
Gender				0.061
Male	93 (85.3%)	16 (14.7%)	109	
Female	127 (76.0%)	40 (24.0%)	167	
Residence				<0.001
Rural	55 (59.8%)	37 (40.2%)	92	
Urban	165 (89.7%)	19 (10.3%)	184	
Training years	1.12 ± 0.44	1.57 ± 0.81	1.22 ± 0.56	0.006

Continuous variables were described using mean (SD).

Categorical variables were described using frequency (percentage).

### Psychological situation of residents

Compared to the Chinese general population, resident physicians showed significant differences on several SCL-90 subscales. Statistically significant differences were observed in obsessive symptoms (*p* < 0.001), depression (*p* = 0.029), anxiety (*p* < 0.001), hostility (*p* < 0.001), terror/phobic anxiety (*p* < 0.001), and paranoia (*p* = 0.007). No significant differences were found for somatization (*p* = 0.171), interpersonal sensitivity (*p* = 0.929), or psychoticism (*p* = 0.307). The results are presented in Table 2.

**Table 2:** Comparison of SCL-90 subscale scores between residents and the Chinese general population.

Subscales	Residents	Chinese general population	*t*	*p*-value
Somatization	1.41 ± 0.48	1.37 ± 0.48	1.373	0.171
Obsessive symptoms	1.81 ± 0.70	1.65 ± 0.50	3.882	<0.001
Interpersonal sensitivity	1.50 ± 0.60	1.50 ± 0.59	0.089	0.929
Depression	1.57 ± 0.67	1.48 ± 0.56	2.199	0.029
Anxiety	1.50 ± 0.55	1.62 ± 0.58	−3.739	<0.001
Hostility	1.36 ± 0.46	1.23 ± 0.41	4.755	<0.001
Terror	1.23 ± 0.39	1.43 ± 0.57	−8.348	<0.001
Paranoia	1.31 ± 0.47	1.39 ± 0.43	−2.711	0.007
Psychosis	1.32 ± 0.46	1.29 ± 0.42	1.022	0.307

### Binary logistic regression results

In our exploratory analysis using binary logistic regression, several factors showed potential associations with psychiatric symptoms after adjusting for multiple comparisons (Table 3). Training years (OR = 2.24, *p* = 0.011) and having a partner (OR = 48.44, *p* < 0.001) were positively associated with psychiatric symptoms. Conversely, residing in an urban area (OR = 0.15, *p* < 0.001), being an only child (OR = 0.35, *p* = 0.020), and holding a physician’s license (OR = 0.15, *p* < 0.001) were associated with a decreased likelihood of psychiatric symptoms. No significant associations were found between psychiatric symptoms and sex (OR = 1.46, *p* = 0.388), training identity (OR = 1.10, *p* = 0.833), education level (OR = 0.63, *p* = 0.285), or household monthly income (OR = 1.01, *p* = 0.888).

**Table 3:** Binary logistic regression of factors associated with psychiatric symptoms.

Variable	OR	95% CI	*p*-value	Adjusted *p*-value
Gender: Female vs. Male (ref: Male)	1.46	(0.62, 3.48)	0.388	0.485
Training years	2.24**	(1.20, 4.17)	0.011	0.029
Residence: Urban vs. Rural (ref: Rural)	0.15***	(0.06, 0.36)	<0.001	<0.001
Partner status: Having partner vs. No partner (ref: No)	48.44***	(9.97, 235.47)	<0.001	<0.001
Only child: Yes vs. No (ref: No)	0.35*	(0.14, 0.85)	0.020	0.041
Physician’s license: Yes vs. No (ref: No)	0.15***	(0.05, 0.41)	<0.001	<0.001
Education: Graduate vs. Bachelor’s degree (ref: Bachelor)	0.63	(0.27, 1.47)	0.285	0.406
Training identity: Professional Master’s degree vs. Others (ref: Others)	1.10	(0.45, 2.68)	0.833	0.888
Household monthly income	1.01	(0.86, 1.18)	0.888	0.888

Results represent exploratory analysis with Benjamini–Hochberg correction for multiple comparisons.

OR, odds ratio; CI, confidence interval. *p < 0.05, **p < 0.01, ***p < 0.001.

## Discussion

This study found that the positive screening rate for psychiatric symptoms among resident physicians, as measured by the SCL-90 scale, was 20.29%, significantly higher than in the Chinese general population. Notably, the subscales of obsessive-compulsiveness, depression, anxiety, hostility, phobic anxiety, and paranoid ideation were all elevated compared to the national general population. These findings are consistent with international research from countries such as the Netherlands and the United States (18, 19), which similarly report elevated psychological distress among resident physicians. These findings can be interpreted through the lens of the Job Demand-Resource (JD-R) model (20), which posits that psychological strain arises when high job demands are not sufficiently counterbalanced by job-related resources. In this context, resident physicians face dual burdens from intense clinical responsibilities and academic obligations while receiving inadequate compensation and support (21). This mismatch between demands and resources may explain the elevated prevalence of psychiatric symptoms observed in this population.

Our exploratory analysis suggested that being in a romantic relationship may potentially be associated with an increased risk of psychiatric symptoms, a counterintuitive result requiring cautious interpretation (22). While earlier studies have often considered partnered status as a protective factor for mental health, our findings suggest otherwise in the context of residency. This discrepancy may be explained by the nature of residency training, which involves prolonged night shifts, emergency duties, and an unpredictable work schedule—circumstances that frequently prevent residents from participating in family life. Such absences may foster disappointment and tension within intimate relationships. Additionally, the generally low income of residents may fall short of their partners’ expectations regarding financial contribution to the household, further exacerbating relational stress.

In our exploratory analysis, the duration of residency training appeared as a potential risk factor, consistent with findings from U.S.-based studies (19). Residency represents a transitional phase in a physician’s career; when compared to age-matched peers in other professions who may have already achieved career stability or advancement, residents may experience heightened feelings of self-doubt and inadequacy.

Conversely, three factors emerged as potential protective factors in this exploratory analysis of psychiatric symptoms: urban residence, only-child status, and holding a physician’s license. These findings align with existing literature and may be explained by several mechanisms. First, residents living in urban areas may have greater access to mental health services, broader social support networks, and a stronger sense of autonomy and personal security—all of which contribute to psychological resilience. Second, individuals with an only-child background may benefit from more concentrated parental attention and emotional support, fostering stronger coping strategies and mental fortitude (23). Third, possession of a physician’s license likely enhances a resident’s sense of professional identity and self-efficacy, providing reassurance regarding their career trajectory and mitigating anxiety about the future.

This study has several limitations. First, due to the cross-sectional design, causal relationships cannot be inferred. Second, the data were based on self-reported questionnaires, which may introduce reporting bias and affect the accuracy of the findings. Third, the study was conducted in a single tertiary hospital, and thus the generalizability of the results may be limited. Future studies should include multi-center evaluations to enhance external validity. Fourth, the multiple regression approach with numerous predictors was inherently exploratory. While we applied false discovery rate correction, the analysis remains susceptible to type I errors and should be considered hypothesis-generating.

## Conclusion

These exploratory findings should be interpreted with appropriate caution. Given the multiple comparisons conducted, the identified associations represent preliminary evidence requiring validation through targeted prospective studies. This study reveals a concerningly high prevalence of psychiatric symptoms among Chinese resident physicians, with significant elevations observed across multiple psychological dimensions. Key risk factors identified include being in a romantic relationship and longer years in training, both of which reflect the compounded pressures of clinical demands, insufficient income, and unmet personal or career expectations. In contrast, urban residence, only-child status, and licensure as a physician appear to confer protective effects, potentially through enhanced access to resources, family support, and a strengthened professional identity. These findings underscore the urgent need for systemic interventions, including improved working conditions in non-urban areas, enhanced emotional and relational support for residents, and structured guidance toward early licensure and career development. Addressing these dimensions holistically may help mitigate mental health risks and promote psychological resilience among medical trainees.

## Data Availability

The raw data supporting the conclusions of this article will be made available by the authors without undue reservation.

## References

[1] Fu X. Survey Report on Mental Health Status of Medical Workers in China in 2020. Beijing: Social Sciences Academic Press (China) (2021). Available at: https://www.pishu.com.cn/skwx_ps/initDatabaseDetail?siteId=14&contentId=12369526&contentType=literature

[2] Nelson J. Physician suicide: investigating its prevalence and cause. Medscape. Available online at: https://www.medscape.com/viewarticle/989674 (Accessed January 7, 2025).

[3] Dyrbye LN Burke SE Hardeman RR Herrin J Wittlin NM Yeazel M. Association of clinical specialty with symptoms of burnout and career choice regret among US resident physicians. JAMA. (2018) 320:1114–30. doi: 10.1001/jama.2018.12615, PMID: 30422299 PMC6233627

[4] Dyrbye LN Thomas MR Massie FS Power DV Eacker A Harper W. Burnout and suicidal ideation among U.S. medical students. Ann Intern Med. (2008) 149:334–41. doi: 10.7326/0003-4819-149-5-200809020-00008, PMID: 18765703

[5] Jackson ER Shanafelt TD Hasan O Satele DV Dyrbye LN. Burnout and alcohol abuse/dependence among U.S. medical students. Acad Med. (2016) 91:1251–6. doi: 10.1097/ACM.0000000000001138, PMID: 26934693

[6] Jaulin F Nguyen DP Marty F Druette L Plaud B Duret C. Perceived stress, anxiety and depressive symptoms among anaesthesia and intensive care residents: a French national survey. Anaesth Crit Care Pain Med. (2021) 40:100830. doi: 10.1016/j.accpm.2021.100830, PMID: 33744493

[7] Liu RQ Davidson J Van Hooren TA Van Koughnett JAM Jones S Ott MC. Impostorism and anxiety contribute to burnout among resident physicians. Med Teach. (2022) 44:758–64. doi: 10.1080/0142159X.2022.2028751, PMID: 35104192

[8] West CP Tan AD Habermann TM Sloan JA Shanafelt TD. Association of resident fatigue and distress with perceived medical errors. JAMA. (2009) 302:1294–300. doi: 10.1001/jama.2009.1389, PMID: 19773564

[9] Civantos AM Byrnes Y Chang C Prasad A Chorath K Poonia SK. Mental health among otolaryngology resident and attending physicians during the COVID-19 pandemic: national study. Head Neck. (2020) 42:1597–609. doi: 10.1002/hed.26292, PMID: 32496637 PMC7300862

[10] Dyrbye LN Massie FS Eacker A Harper W Power D Durning SJ. Relationship between burnout and professional conduct and attitudes among US medical students. JAMA. (2010) 304:1173–80. doi: 10.1001/jama.2010.1318 20841530

[11] Spiotta AM Fargen KM Patel S Larrew T Turner RD. Impact of a residency-integrated wellness program on resident mental health, sleepiness, and quality of life. Neurosurgery. (2019) 84:341–6. doi: 10.1093/neuros/nyy112, PMID: 30169852

[12] Ueno T Ito K Murai T Fujiwara H. Mental health problems and their association with internet use in medical residents. Front Public Health. (2020) 8:587390. doi: 10.3389/fpubh.2020.587390, PMID: 33194994 PMC7641600

[13] Dyrbye LN Eacker A Durning SJ Brazeau C Moutier C Massie FS. The impact of stigma and personal experiences on the help-seeking behaviors of medical students with burnout. Acad Med. (2015) 90:961–9. doi: 10.1097/ACM.0000000000000655, PMID: 25650824

[14] Wellness and work: mixed messages in residency training - PubMed. Available online at: https://pubmed.ncbi.nlm.nih.gov/30924087/ (Accessed January 7, 2025).

[15] Real Noval H Martin Parra JI Fernández Fernández J Del Castillo Criado Á Ruiz Gómez JL López Useros A. Sleep deprivation among surgical residents: does it affect performance while practising a laparoscopic intestinal anastomosis? Cirugía Española (English Edition). (2022) 100:223–8. doi: 10.1016/j.cireng.2022.03.014, PMID: 35431159

[16] Cole JO. Psychopharmacology Bulletin The Clearinghouse (1966).

[17] Hou T Zhang T Cai W Song X Chen A Deng G. Social support and mental health among health care workers during coronavirus disease 2019 outbreak: a moderated mediation model. PLoS One. (2020) 15:e0233831. doi: 10.1371/journal.pone.0233831 32470007 PMC7259684

[18] Baas MAM Stramrood CAI Molenaar JE van Baar PM Vanhommerig JW van Pampus MG. Continuing the conversation: a cross-sectional study about the effects of work-related adverse events on the mental health of Dutch (resident) obstetrician-gynaecologists (ObGyns). BMC Psychiatry. (2024) 24:286. doi: 10.1186/s12888-024-05678-3, PMID: 38627649 PMC11022402

[19] Chen L Zhao Z Wang Z Zhou Y Zhou X Pan H. Prevalence and risk factors for depression among training physicians in China and the United States. Sci Rep. (2022) 12:8170. doi: 10.1038/s41598-022-12066-y, PMID: 35581251 PMC9112267

[20] Demerouti E Bakker AB Nachreiner F Schaufeli WB. The job demands-resources model of burnout. J Appl Psychol. (2001) 86:499–512. doi: 10.1037/0021-9010.86.3.499 11419809

[21] Yu X Zheng F. Analysis of mental health status and influencing factors of general practitioner resident trainees in standardized training: a mixed-method study. Gen Pract China. (2022) 25:2028–35.

[22] Gallagher EJ Conlin PR Kazmierczak BI Vyas JM Ajijola OA Kontos CD. Is it time to reduce the length of postgraduate training for physician-scientists in internal medicine? JCI Insight. (2024) 9:e178214. doi: 10.1172/jci.insight.178214, PMID: 38775155 PMC11141926

[23] Jiang Y. Implicit personality, psychological resilience and emotional regulation among college students. ResearchGate. (2025). Available online at: 10.5861/ijrse.2025.25609

